# Analysis of Media Outlets on Women's Health: Thematic and Quantitative Analyses Using Twitter

**DOI:** 10.3389/fpubh.2021.644284

**Published:** 2021-05-31

**Authors:** Miguel A. Alvarez-Mon, Carolina Donat-Vargas, Maria Llavero-Valero, Alfredo Gea, Melchor Alvarez-Mon, Miguel A. Martinez-Gonzalez, Cristina Lopez-del Burgo

**Affiliations:** ^1^Department of Psychiatry and Medical Psychology, Hospital Universitario Infanta Leonor, Madrid, Spain; ^2^Department of Medicine and Medical Specialities, Faculty of Medicine and Health Sciences, University of Alcala, Alcalá de Henares, Spain; ^3^Cardiovascular and Nutritional Epidemiology, Institute of Environmental Medicine, Karolinska Institute, Stockholm, Sweden; ^4^Unit of Nutritional Epidemiology, Institute of Environmental Medicine, Karolinska Institutet, Solna, Sweden; ^5^IMDEA-Food Institute, CEI UAM+CSIC, Madrid, Spain; ^6^Service of Endocrinology and Nutrition, Infanta Leonor Hospital, Madrid, Spain; ^7^Department Preventive Medicine and Public Health, School of Medicine, University of Navarra, Pamplona, Spain; ^8^Center for Biomedical Research in the Liver and Digestive Diseases Network, Madrid, Spain; ^9^Service of Internal Medicine and Rheumatology/Autoimmune Diseases, Príncipe de Asturias University Hospital, Alcalá de Henares, Spain; ^10^Ramón y Cajal Institute for Health Research, Madrid, Spain; ^11^Department of Nutrition, School of Public Health, Harvard University, Cambridge, MA, United States; ^12^Instituto de Investigación Sanitaria de Navarra (IdiSNA), Pamplona, Spain; ^13^Institute for Culture and Society, University of Navarra, Pamplona, Spain

**Keywords:** hormonal contraception, birth control, twitter, women's health, osteoporosis, bone health

## Abstract

**Background:** Media outlets influence social attitudes toward health habits. The analysis of tweets has become a tool for health researchers.

**Objective:** The objective of this study was to investigate the distribution of tweets about women's health and the interest generated among Twitter users.

**Methods:** We investigated tweets posted by 25 major U.S. media outlets about pre-menopausal and post-menopausal women's health between January 2009 and December 2019 as well as the retweets generated. In addition, we measured the sentiment analysis of these tweets as well as their potential dissemination.

**Results:** A total of 376 tweets were analyzed. Pre-menopausal women's health accounted for most of the tweets (75.3%). Contraception was the main focus of the tweets, while a very limited number were related to infertility (1.4%). With regard to medical content, the effectiveness of contraceptive methods was the most frequent topic (46.2%). However, tweets related to side effects achieved the highest retweet-to-tweet ratio (70.3). The analysis of sentiments showed negative perceptions on tubal ligation.

**Conclusions:** The U.S. media outlets analyzed are more interested in pre-menopausal than in post-menopausal women health and focused their content on contraception, while Twitter users showed greater interest in side effects.

## Introduction

Over the last decade, the internet, and particularly social media, have modified how people find, communicate, and share information with one another and the rest of the world (1). Social media has become ubiquitous, with more people accessing web-based medical content by following links on social media than through direct searches (2). As a result, most of the media outlets have exponentially increased the dissemination of information through social media, particularly through Twitter (3). These media outlets use tweets as a tool for news distribution. As these messages have an important influence on lifestyles and health behaviors, they therefore are important from a public health perspective.

Social media platforms like Twitter are increasingly being leveraged by researchers for public health surveillance, intervention delivery, the study of attitudes toward health behaviors and diseases, predictions on the incidence of both communicable and non-communicable diseases, and insight into the medical experiences of patients (4, 5). In this context, the feasibility of using social media to study mass media outlet's content and social perceptions regarding diseases has been sufficiently established (6).

The interest of society in health conditions and diseases is determined by different factors, including medical parameters such as their prevalence, morbidity, and mortality, as well as advancements in their diagnosis, prevention, and treatment (7, 8). The number and characteristics of the potential individual targets of the disease is also a relevant factor (9). Women represent roughly one half of the global population and have specific health conditions, including a susceptibility to highly prevalent diseases (10). For pre-menopausal women, fertility and pregnancy are among the most important health concerns (11). In fact, birth control has become a priority among global public health agendas (12). The potential use of different options of contraception is a matter of personal choice, based on different factors including access to relevant medical information (13). Though combined hormonal contraceptives (CHCs) are known to reduce the risk of ovarian cancer, CHCs increase the risks of severe chronic disease, including cardiovascular disease and breast cancer, which are prominent causes of death among women (14, 15). These facts, together with the finding of raised risks of venous thromboembolism and pulmonary embolism with third-generation CHCs, have increased concerns about the side effects of CHCs among women of childbearing age (16, 17). At the same time, the prevalence of infertility is increasing and is a worrisome problem (18). Additionally, in post-menopausal women, bone health is recognized as a common health concern, with osteoporosis being the most prevalent and preventable severe bone disease (19). Furthermore, sexual dysfunction is another common health problem, especially in midlife women, with a significant negative impact on quality of life (20, 21).

The current study seeks to undertake the following objectives: first, to determine the themes emerging from U.S. mass media tweets about pre-menopausal (contraception, infertility, and sexual dysfunction) and post-menopausal (osteoporosis, prevention, and treatment) women's health; second, to investigate the interest generated by these tweets within the Twitter community; third, to examine the dissemination of these tweets on Twitter; and fourth, to describe how media coverage of women's health-related tweets has changed over time. To address these questions, we analyzed a large-scale set of tweets posted by 25 major U.S. media outlets throughout a decade concerning hashtags covering contraception, infertility, bone health, and sexual dysfunction.

## Materials and Methods

### Search Strategy

We focused our study on tweets concerning pre- and post-menopausal women's health posted by a sample of 25 major U.S. media outlets beginning on January 2009 and concluding on December 2019. We included 11 newspapers (*The New York Times, Washington Post, Los Angeles Times, USA Today, The Chicago Tribune, New York Post, Wall Street Journal, New York Daily News, Boston Globe, San Francisco Chronicle*, and British *Daily Mail*), six broadcast network television or cable news sites (MSNBC, CNN, ABC News, Fox News, CBS News, and BBC News), one wire service news site (Reuters), three hybrid online-only sites (Yahoo news, AOL News, and Huffington Post), and four pure news aggregators (Google News, The Examiner, Topix, and Bing News). These media outlets, which are among the most influential media outlets in the USA (although the headquarters of some of them are based in other countries), were selected based on their number of followers on Twitter, as shown by their individual accounts and their social influence during the time of the study (22).

### Collection of Twitter Data

Our research strategy focused on searching for tweets referring to contraception, infertility, sexual dysfunction, or bone health. We investigated all tweets posted from the Twitter accounts of the previously mentioned media outlets, filtering them according to specific criteria using the following list of hashtags: #Nuvaring, #Femcap, #Diaphragm, #Intrauterinedevice, #Mirena, #Liletta, #Kyleen, #Skyla, #Paragard, #sponge, #Tuballigation, #Vasectomy, #Nexplanon, #Implanon, #Depro-provera, #LactationalAmenorrheaMethod, #FamilyAwareness Methods, #Temperaturemethod, #Cervicalmucusmethods, #Calendarmethod, #Parche, #Xulane, #Birthcontrolpills, #combinedestrogenprogestin, #oralcontraceptives, #Alesse, #Lessina, #Mircette, #Contraception, #Infertility, #Sexualdysfunction, #Osteopenia, #Osteoporosis, #Forteo, #Teriparatide, #Prolia, #Denosumab, #Bisphosphonates, #Alendronate, #Binosto, #Fosamax, #Ibandronate, #Boniva, #Risedronate, #Actonel, #Atelvia, #Zoledronicacid, #Reclast, and #Zometa.

We grouped the hashtags into two categories: pre-menopausal- and post-menopausal-related hashtags. In addition, we divided the pre-menopausal group into three categories: (1) contraception-related hashtags (including surgical, non-surgical, and natural family planning methods); (2) sexual dysfunction; and (3) infertility. The post-menopausal group was divided into two categories: (1) bone health (including osteoporosis, osteopenia, and treatment related tweets) and (2) sexual dysfunction. The inclusion criteria for tweets were as follows: (1) being posted by any of the 25 U.S. media outlets selected for our study; (2) using any of the previously mentioned hashtags; (3) being posted between January 1, 2009, and December 31, 2019; and (4) being written in English. The 11-year period was chosen to align our research with the past decade. We excluded tweets that provided information that was too limited (e.g., tweets consisting mainly of hashtags) or contained only images.

### Content Analysis Process

All 477 retrieved tweets were directly inspected by two raters (ML-V and MAA-M). First, all of the tweets were scanned, in order to identify the most common tweet themes. Secondly, a codebook was created based on the research questions, our previous experience in analyzing tweets, and the most common themes. Third, the two raters analyzed the tweets separately, and discrepancies were discussed between the raters and a senior author (MA-M). We excluded 101 tweets that provided information that was too limited or included content not related to our research questions. This process led to the creation of a more concise database, which yielded a total of 376 tweets. All remaining tweets were considered classifiable and were coded according to our codebook. Each tweet, depending on the content, was rated as medical (if it included a reference to efficacy, side effects, or other medical related facts) or non-medical (business, legal, or other contents). The coding categories were mutually exclusive. In the case content was found to be almost identical in different tweets, it was classified in the same way as the first tweet encountered. The classification criteria we used and examples of tweets according to each category are shown in Tables 1, 2.

**Table 1:** Definitions of the medical categories and examples.

**Categories**	**Examples of tweets**
Effectiveness (refers to the ability or inability of a contraceptive method to prevent an unplanned pregnancy)	• IUDs are more than 99% effective in preventing pregnancy, and some can work up to 12 years, but a 2016 survey found that many women still do not know anything about these longer-acting reversible contraceptives • Pediatricians call IUDs “first-line” birth control for teens • The IUD is 20 times more effective than birth control pills, as studies have found • <1% of women who use an IUD become pregnant in 1 year, compared with 9% of women who use birth control pills
Side effects (refer to any effect that is secondary to the one intended; we also included tweets discussing tolerability of the drug)	• This form of IUD may carry a higher risk for breast cancer in certain women • A large study of Danish women finds that birth control pills and IUDs that release hormones carry a risk for breast cancer • Women taking newer forms of birth control pills have higher risk of blood clots, as studies have shown • Teenage girls who use birth control pills are more likely to cry, sleep too much, and experience eating issues than their peers who do not use oral contraceptives, according to a recent study published in the medical journal *JAMA Psychiatry*. • “20–30% of women on birth control pills experience depression. This study was terminated after 3% depression in men” • A British woman is dead after developing a blood clot caused by birth control pills
Other	• #Doh, Not Again … “We forgot the birth control pills” -Levi Johnston on prego girlfriend • Do women on birth control pills prefer men with less masculine facial features?

*IUD, intrauterine device*.

**Table 2:** Definitions of the non-medical categories and examples.

**Categories**	**Examples of tweets**
Legal issues	• More than 1,000 suits against NuvaRing to go to trial this fall • Merck to pay $100 million in NuvaRing settlement • Inmates in one Tennessee county can reduce jail time with birth control—implant for females and vasectomy for males • Virginia man agrees to vasectomy to reduce prison sentence • Pregnant women sue pharmaceutical companies over mislabeled birth control pills
Economic aspects	• Teva to sell contraceptive brand ParaGard in $1.1 billion deal • Pfizer recalls a million packets of birth control pills
Other	• Ron Paul: “The [birth control] pills can't be blamed for the immorality of our society” • Startups that deliver birth control pills, other meds @XXX @XXX via @XXX

*Usernames and personal names were replaced by XXX*.

### Search Tool Used

In this study, we used Twitter Firehose operated by GNIP, which allows access to 100% of all public tweets that match some sort of “search” criteria (query). Additionally, the search criteria used were the previously indicated hashtags. Tweet Binder, the search engine we employed, uses automatic machine learning text analysis algorithms. Tweet Binder uses node.js and PHP language, which enables the analysis of tweets in json format (used by GNIP).

After the tweets were obtained from Tweet Binder, the data were imported to Microsoft Excel spreadsheets. The username, the date and time tweeted, the complete text of the tweet, the permanent link to the tweets, and the number of retweets and likes generated by each tweet were collected. In addition, the number of followers and the number of tweets posted by each mass media outlet for said hashtags were registered.

### Measuring Attitudes and Influence on Twitter

We analyzed the number of retweets and likes generated by each tweet as an indicator of user interest in a given topic. We also measured the potential reach of all analyzed hashtags in order to best assess tendencies in the dissemination of tweets. For the purpose of this study, reach is defined as a numerical value measuring the potential audience of the hashtag (how many Twitter users may potentially see it). To calculate the reach of each hashtag, we added all the followers of each Twitter user (the Twitter account of the 25 major U.S. media outlets selected) who participated in the hashtag.

The potential reach and impact of all analyzed hashtags were measured to best assess tendencies in the dissemination of tweets, and by sentiment analysis, we classified the polarity of the tweet—whether the expressed idea has a positive, negative, or neutral connotation. Sentiment analysis tools analyze all the words contained in each tweet, and each word has its own score that can vary depending on the context. The average score of all the tweets with a certain hashtag determined the overall sentiment score. According to that score, we classified each hashtag into three categories: negative (0–40), neutral (>40–60), and positive (>60–100). The search engine Tweet Binder provided these data.

### Ethical Considerations

This study received the approval of the University of Alcala Research Ethics Committee and was compliant with the research ethics principles of the Declaration of Helsinki (7th revision, 2013). However, this study did not directly involve human subjects nor include any interventions, instead using publicly available tweets.

### Statistical Analysis

The series of tweets were statistically analyzed to describe the number of tweets, retweets, and likes per treatment category and subcategory, considering retweets and likes as indices for reflecting the users' interests in the particular treatment. We also calculated the ratio retweet/tweet to provide further information. The statistical significance of the difference between the number of tweets generated with medical content and non-medical content was calculated using the Pearson chi-squared test (statistical significance was set at two-sided *P* < 0.05). Further, we visually displayed the temporal trend over 10 years of tweets, retweets, and likes of pre-menopause (birth control) and compared the temporal trend of tweets between pre- and post-menopause treatments. These analyses and graphs were conducted with the software packages STATA v16 (StataCorp) and Microsoft Excel (Windows).

## Results

### Contraception Is the Focus of the Women's Health-Related Tweets Generated by Major U.S. Media Outlets

When we investigated the women's health-related tweets posted by 25 major U.S. media outlets from January 2009 through December 2019 (with hashtags related to contraception, infertility, sexual dysfunction, and bone health), of the total of 49 hashtags analyzed, only 13 (26.5%) generated at least one tweet: NuvaRing, intrauterine device (IUD), ParaGard, tubal ligation, vasectomy, Nexplanon, birth control pills (BCPs), contraception, and infertility in the pre-menopausal group; and osteoporosis, Fosamax, and Boniva in the post-menopausal group.

A total of 376 tweets about women's health were analyzed (Table 3). Among them, 283 (75%) tweets were included in the pre-menopausal group and 93 (25%) in the post-menopausal group. The number of tweets relating to each of the categories of birth control followed a heterogeneous pattern of distribution. Contraception-related tweets accounted for almost all of the tweets (98.6%), whereas tweets related to infertility only accounted for 1.4%. Additionally, 96.8% of the tweets in the contraception group were specifically focused on hormonal contraception, IUD, and vasectomy. Of note, natural family planning methods did not generate tweets. In the post-menopausal group, all of the tweets were related to bone health. Moreover, sexual dysfunction did not generate any tweets in either group.

**Table 3:** Number of tweets and retweets generated in pre-menopause and post-menopause.

	**Tweets**		**Retweets**		**Ratio retweet/tweet**
	***n***	**%**	***n***	**%**	
**Pre-menopause**					
**Contraception**					
No surgical					
Hormonal contraception^*^	97	34.3	6,050	42.29	62.37
Specific hormonal contraceptives^†^	6	2.1	88	0.62	14.67
Intrauterine device (IUD)	73	25.8	4,860	33.97	66.58
Surgical therapy					
Tubal ligation	3	1.1	29	0.20	9.67
Vasectomy	100	35.3	3,072	21.48	30.72
Natural family planning	–	–	–	–	
**Sexual dysfunction**	–	–	–	–	
**Infertility**	4	1.4	206	1.44	51.50
**Total**	283	100	14,305	100	50.55
**Post-menopause**					
**Bone health**					
Osteoporosis (disease)	75	80.6	2,232	90.8	29.76
Osteoporosis prevention/treatment	18	19.4	227	9.2	12.61
**Sexual dysfunction**	–	–	–	–	
**Total**	93	100	2,459	100	26.44

*Percentages (%) were calculated with respect to the total number of tweets distributed within the pre-menopause and post-menopause groups and the retweets generated*.

* *Hormonal contraception includes contraception and birth control pills*.

† *Specific hormonal contraceptives include Nexplanon, NuvaRing, Sponge, and ParaGard*.

Next, we analyzed the medical and non-medical contents of the women's health-related tweets that we studied (Table 4). We found a significant difference in the percentage of tweets with medical and non-medical contents between the different categories analyzed. In the pre-menopausal women's health group, the percentage of tweets with medical content was higher among those related to IUD (78.1%) and hormonal contraception (55.7%). In contrast, non-medical content was higher (59%) in vasectomy-related tweets. In the post-menopausal group, 93.3% of osteoporosis and osteopenia-related tweets had medical content, whereas only 16.6% of the tweets containing content related to treatment or preventive measures had a medical content.

**Table 4:** Number of tweets generated in pre-menopause and post-menopause classified by medical and non-medical content.

	**Tweets**					
	**Medical**		**Non-medical**		**Total**	
	***n***	**%**	***n***	**%**	***n***	**%**
**Pre-menopause**						
**Contraception**						
No surgical						
Hormonal contraception^*^	54	55.67	43	44.33	97	100
Specific hormonal contraceptives^†^	1	16.67	5	83.33	6	100
Intrauterine device (IUD)	57	78.08	16	21.92	73	100
Surgical therapy						
Tubal ligation	1	33.33	2	66.67	3	100
Vasectomy	41	41.00	59	59.00	100	100
Natural family planning	–	–	–	–	–	–
**Sexual dysfunction**	–	–	–	–	–	–
**Infertility**	4	100.00	0	0.00	4	100
Chi^2^ *P*-value					<0.001	
**Post-menopause**						
**Bone health**						
Osteoporosis (disease)	70	93.33	5	6.67	75	100
Osteoporosis prevention/treatment	3	16.67	15	83.33	18	100
**Sexual dysfunction**	–	–	–	–	–	–
Chi^2^ *P*-value					<0.001	

*Percentages (%) were calculated with respect to the total number of tweets distributed within the pre-menopause and post-menopause groups. A chi-square test is used to see if the number of tweets for each treatment differs when they have medical or non-medical content*.

* *Hormonal contraception includes contraception and birth control pills*.

† *Specific hormonal contraceptives include Nexplanon, NuvaRing, Sponge, and ParaGard*.

We also analyzed the areas of interest in the medical and non-medical content of those tweets related to pre-menopausal and post-menopausal women's health (Table 5). In the pre-menopausal group, the medical content was mainly focused on the effectiveness of the procedure or treatment, while the non-medical content was focused on legal aspects. In the post-menopausal group, the medical content of the tweets was distributed between the pathogenesis and diagnosis of the disease and its treatment.

**Table 5:** Retweet/tweet ratios in pre-menopause (birth control) and post-menopause (osteoporosis).

	**Tweet (*****n*****)**		**Retweet (*****n*****)**		**Ratio retweet/tweet**
**Pre-menopause (birth control)**	***n***	**%**	***n***	**%**	
**Medical content**	158	100	7,920	100	50.1
Effectiveness	73	46.2	3,306	41.7	45.3
Side effects	49	31.0	3,446	43.5	70.3
Other	36	22.8	1,168	14.7	32.4
**Non-medical content**	125	100	6,385	100	51.1
Legal issues	78	62.4	4,997	78.3	64.1
Economic aspects	10	8.0	252	3.9	25.2
Other	37	29.6	1,136	17.8	30.7
**Post-menopause (osteoporosis)**	***n***	**%**	***n***	**%**	
**Medical content**	73	100	2,172	100	29.8
Osteoporosis/osteopenia	37	50.7	904	41.6	24.4
Preventive treatment	36	49.3	1,268	58.4	35.2
Other	0	0.0	0	0.0	
**Non-medical content**	20	100	287	100	14.4
Legal issues	18	90.0	286	99.7	15.9
Economic aspects	2	10.0	1	0.3	0.5
Other	0	0.0	0	0.0	0

### Side Effects of Birth Control Methods Generated the Highest Response Among the Twitter Community

We investigated the interest in tweets posted by U.S. media outlets among social media users by analyzing the number of retweets received (Table 3). We observed a correlation between the number of tweets posted referring to each different category and the number of subsequent retweets. Furthermore, there was a significant correlation between the number of retweets and likes generated by the tweets of each different category. We also investigated the retweet-to-tweet ratio for the tweets of each different category. The retweet-to-tweet ratio of hormonal contraception (62.37) and IUD (66.58) tweets was more than twice the number of vasectomy-related tweets (30.72). Interestingly, the reduced number of infertility-related tweets showed an elevated retweet-to-tweet ratio (51.50). Finally, the retweet-to-tweet ratio of the tweets related to pre-menopausal women's health (50.55) was higher than that related to post-menopausal women's health (26.44).

Next, we analyzed the retweet-to-tweet ratio of the tweets generated by each different category classified according to their medical or non-medical content (Table 5). There were differences in the retweet-to-tweet ratio between the tweets classified according to their medical or non-medical content. In the pre-menopausal women's health group, we found that the content of both medical and non-medical tweets had a similar retweet-to-tweet ratio. In contrast, in the post-menopausal women's health group, tweets with medical content had higher retweet-to-tweet ratios than those with non-medical content. The highest retweet-to-tweet ratio was observed in the tweets related to side effects of the contraception methods (70.3) followed by those related to the legal aspects of these procedures (64.1).

### Throughout the Last Decade, U.S. Media Outlets Have Been More Interested in Pre-menopausal than in Post-menopausal Women's Health, and a High Interest in Side Effects of Contraception Methods Is Apparent

**Figure 1:**
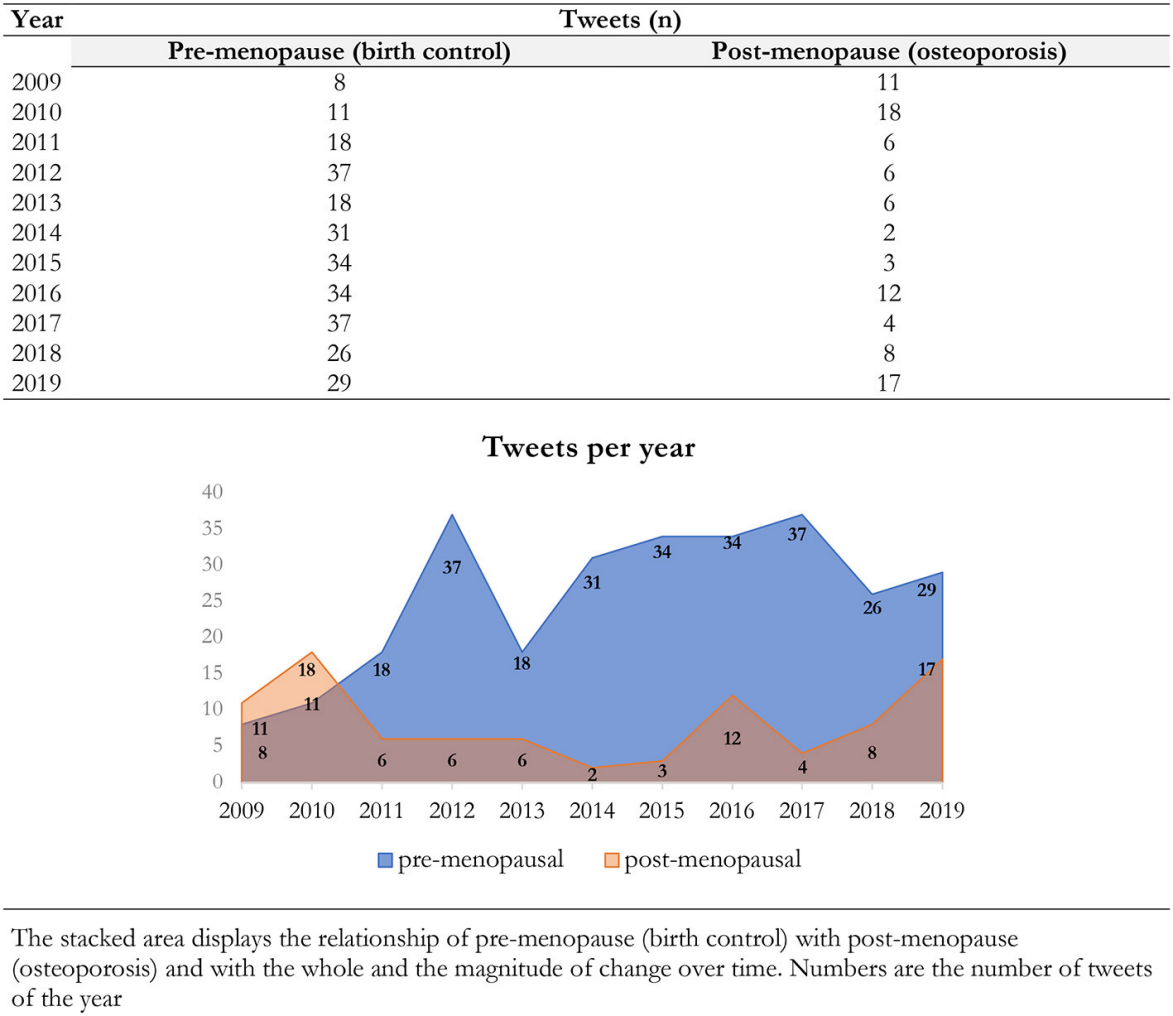
Comparison of the temporal trend over 11 years of tweets between pre-menopause (birth control) and post-menopause (osteoporosis) treatments. The stacked area displays the relationship of pre-menopause (birth control) with post-menopause (osteoporosis) and with the whole and the magnitude of change over time. Numbers are the number of tweets of the year.

We assessed the evolution of the number of tweets related to pre-menopausal and post-menopausal women's health posted by U.S. media outlets throughout the 11 years of our study (Figure 1). The total number of tweets posted about women's health remained fairly constant over the last 8 years of the study. With the exception of the first 2 years of the decade analyzed, the number of tweets posted about pre-menopausal women's health was higher than that about post-menopausal women health. Interestingly, however, a recent increase in those tweets related to post-menopausal women's health has been observed since 2016.

**Figure 2:**
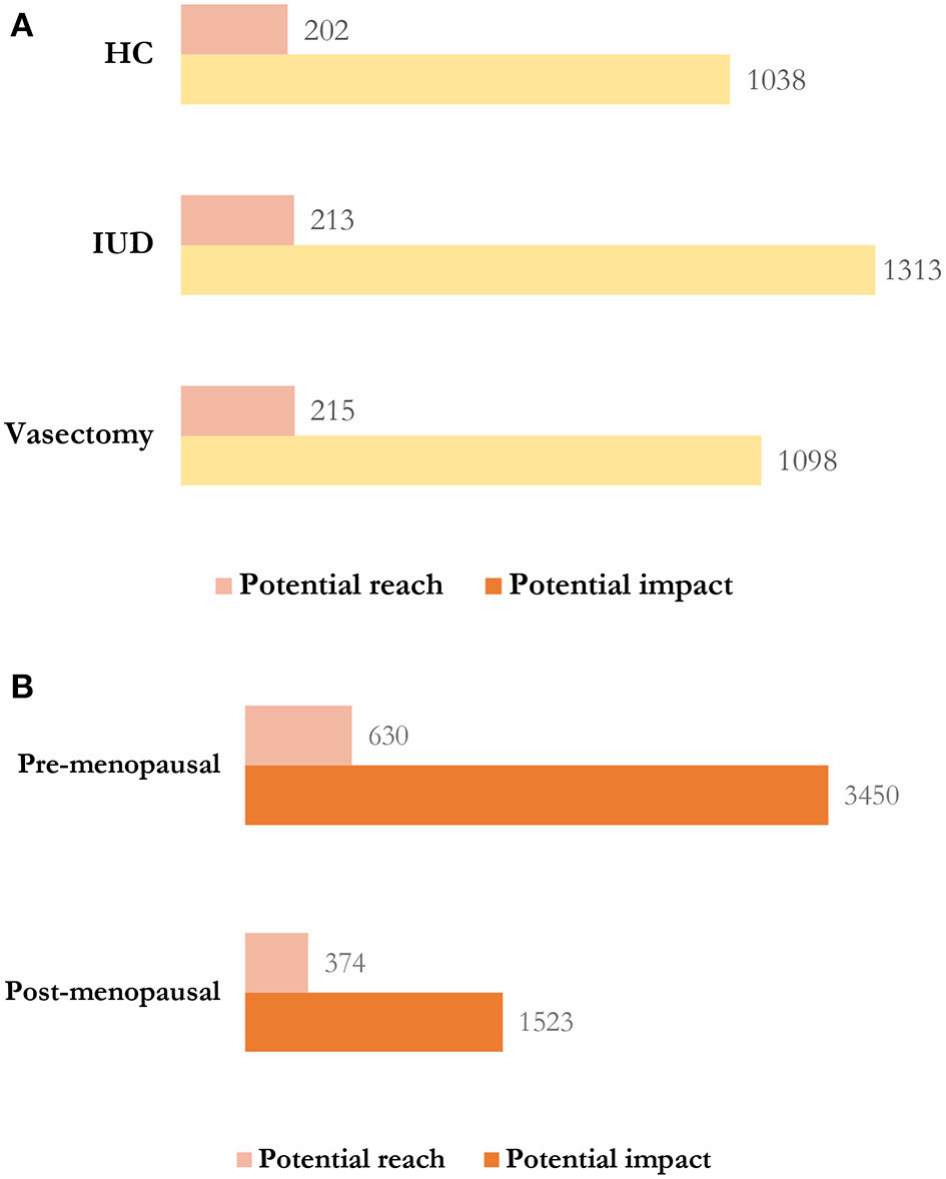
**(A)** Potential impact and potential reach of the pre-menopause tweets (in millions). HC, hormonal contraception; IUD, intrauterine device. **(B)** Potential impact and potential reach comparisons between pre-menopause and post-menopause treatments (in millions).

We also studied the tendencies in the dissemination of tweets posted about women's health. We studied the potential impact and reach of the tweets generated about pre-menopausal and post-menopausal women's health (Figure 2). In doing so, we found that the potential impact of the tweets related to pre-menopausal women's health doubled that of post-menopausal women's health. Likewise, similar differences were observed in the potential reach of those tweets related to both pre- and post-menopausal women's health.

**Figure 3:**
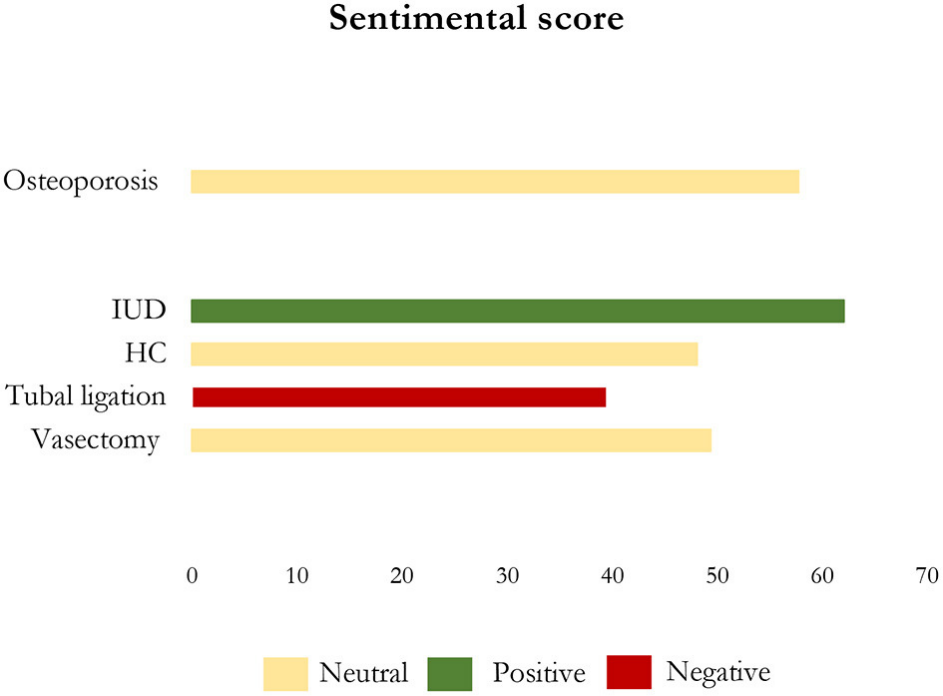
Sentimental score comparisons between pre-menopause and post-menopause treatments. IUD, intrauterine device; HC, hormonal contraception. Sentiment analysis is classifying the *polarity* of the tweet—whether the expressed idea has positive, negative, or neutral connotation.

Finally, we analyzed the sentiment score of the different hashtags (Figure 3). We found that those posts related to IUD received positive sentiments, while those posts related to tubal ligation received a negative sentiment. However, the sentiment was neutral for osteoporosis, hormonal contraception, and tubal ligation.

## Discussion

### Principal Findings

In this study, according to the tweets posted, we have found that the main U.S. media outlets are more interested in pre-menopausal women's health than in post-menopausal women health. In terms of content, contraception was the main focus of the posted tweets, while a very limited number were related to infertility. However, the latter group of tweets generated a large response among Twitter users. With regard to medical content, the effectiveness of contraceptive methods was the most frequent topic; however, those tweets related to side effects of contraceptive methods accounted for the maximum level of responses within the Twitter community. The post-menopausal-related tweets clearly showed a lesser reach and impact, as well as a reduced response, in comparison with those tweets related to pre-menopausal women's health among Twitter followers. Finally, sexual dysfunction and natural family planning failed to receive any tweets.

Nowadays, the relevant role of media outlets in generating popular opinion, perceptions, and emotions via the spread of information is readily recognized (23). In addition to traditional forms of communication, both the internet and social media have become particularly pivotal instruments for distributing knowledge and news (24). Likewise, Twitter is currently considered an equally effective channel for communication (25). We have found that the tweets posted by 25 major U.S. media outlets about women's health were mainly focused on pre-menopausal-related aspects. More specifically, the principal area of interest was contraception, whereas infertility was the subject of only a limited number of tweets, and sexual dysfunction was void of any references. This heterogeneous distribution of interest shown by the U.S. media outlets may be explained in part by the significance of the potential for pregnancy in American women. However, infertility is a relatively common health condition affecting an estimated 12–18% of couples (26). Interestingly, the limited quantitative relevance given by U.S. media outlets to infertility differs from that observed in diseases or health conditions such as diabetes or depression that have a similar prevalence (6). Furthermore, the diagnosis and treatment of infertility are areas of great medical interest and have required their own specialized fields in health care (27). The scarcity of content related to infertility may be explained by the fact that we have limited our research to news posted in Twitter. In addition, another potential explanation is that we might have missed some tweets related to infertility because we did not include other related terms different from “infertility,” such as tubal blockage, pelvic inflammatory disease, endometriosis, or ovulation problems. Regarding sexual dysfunction in women, even though it occurs in an estimated 40% of pre-menopausal women, we have not encountered any tweets posted by the U.S. media outlets about this medical condition (21).

The areas of interest in women's contraception are mainly focused on non-surgical methods (hormonal methods and IUD), while there has been limited interest generated in surgical female sterilization (tubal ligation). Interestingly, several studies have demonstrated that hormonal contraceptives are associated with harmful side effects such as higher risk of breast cancer, myocardial infarction, and ischemic stroke or higher risk of obesity development, among others (14, 15, 28). Surgical methods, on the other hand, are associated with less detrimental effects. Tubal ligation is quite commonly used as a birth control method, but it obtained the lowest retweet/tweet ratio as well as the lowest sentiment score. These findings suggest that a permanent method of sterilization, such as tubal ligation, is less valued among Twitter users who in general tend to be younger than the overall population.

On the other hand, male sterilization-related tweets (vasectomy) accumulated a third of the contraception tweets. Additionally, the distribution of tweets related to contraceptive methods posted by the U.S. media outlets poorly correlates with those currently used by the U.S. population at large (29). It is noteworthy that natural family planning has not received any interest from these outlets despite its high effectiveness, low cost, and lack of side effects (30, 31). The absence of interest on these methods may represent a potential drawback for public health, given their advantages (32). A likely explanation for that lack of attention (reflecting scarce knowledge) might be that no pharmaceutical companies have a commercial interest in the promotion of fertility-awareness methods.

For post-menopausal women, bone health is considered a significant area of prevention and medical care (33). In this regard, osteoporosis is one of the most prevalent and severe diseases affecting this group (19). However, the number of tweets posted by U.S. media outlets about bone health and osteoporosis has been limited as compared with that observed with respect to pre-menopausal women's health. Interestingly, osteoporosis and its treatment received a very reduced number of tweets in comparison with diseases affecting women or men of a similar age range such as Parkinson (6). For example, breast cancer has received a significantly higher number of tweets from U.S. media outlets over a similar period of time (6). It is therefore possible to consider that the limited number of new treatments developed to combat osteoporosis in the previous decade might be responsible for the observed limited interest in the disease. Furthermore, sexual dysfunction, despite being a common health condition in post-menopausal women, did not receive any tweets from U.S. media outlets throughout the decade of our study (21). It could be that this topic is still “taboo,” especially among older women, finding it difficult to talk openly about it. Another reason for not finding tweets about this topic could be the use of the term “sexual dysfunction.” Women experiencing dyspareunia, low libido, or lack of orgasms may use more colloquial hashtags. Taken together, the interest in tweets regarding women's health shown by the U.S. media outlets appears not to be linked to the prevalence of health-related behaviors nor to the prevalence of medical conditions.

In addition to the quantitative study of the tweets, content analysis has also been a pivotal tool for infodemiology (34). For pre-menopausal women's health, the content of the tweets posted by U.S. media outlets was slightly more focused on medical features than on non-medical aspects. However, it was also shown that this same tendency was higher in those tweets related to IUD than in those related to hormonal contraception. Of note, in regard to medical content, the effectiveness of contraceptive methods accounted for the highest number of posted tweets, whereas those tweets addressing side effects generated the most interest from among Twitter users, as evidenced by their having attained the highest retweet-to-tweet ratio. Accordingly, it has been reported that the side effects of contraceptive methods are a matter of significant concern for female users (35, 36). It is additionally relevant to indicate that this ratio is similar to that found for tweets posted by U.S. media outlets on diseases with a high degree of prevalence and morbidity (6). These findings may be related to the key role that efficacy and side effects play in terms of birth control for female users. Regarding contraception, tweets with non-medical content were predominated by those related to vasectomy and were mainly focused on legal aspects. Interestingly, sterilization in men has important legal considerations and associated social controversies (37). Notably, the content of posted tweets about osteoporosis treatment was mainly non-medical and focused on legal matters. In this regard, it is worth highlighting the media attention generated by a specific drug-related scandal (38). This finding suggests an uncommon bias toward the medical treatment of osteoporosis that has not been previously directed at other drugs and diseases.

Our data show that the number of tweets sent by 25 major U.S. outlets about pre-menopausal women's health has remained fairly stable over the last 8 years of the study, but a recent increase in those tweets related to post-menopausal women's health has been observed since 2016. Nonetheless, U.S. outlets have overall been more interested in physical and mental diseases than in women's health, as previously reported in another study that analyzed the tweets sent by 15 major U.S. media outlets during one decade (6). The absence of interest shown by major U.S. outlets toward pre- and post-menopausal sexual dysfunction is surprising given previous reports of great interest in anxiety and depression (6). However, the interest stemming from women's health-related tweets sent out by media outlets, as measured by the number of retweets generated by followers, is clearly relevant. More specifically, the retweet frequency is a parameter that indicates user interest in the topic of each tweet (39). Herein, our data demonstrate that the retweet-to-tweet ratio generated by pre-menopausal women's health-related tweets was higher than that of post-menopausal women's health. Several reasons may explain this observed difference in responses generated among Twitter users, but the reported younger age of Twitter users might play a significant role in explaining this fact (40). In addition, the content of the tweets may also be involved in these distinct responses since the lowest retweet-to-tweet ratio was found in tweets with non-medical post-menopausal women's health content.

Although our results suggest that women's health is not a relevant area of interest for U.S. media outlets in terms of their Twitter communication, medical content nevertheless prevailed over non-medical content among the posted tweets. These results are promising due to the existence of evidence supporting Twitter as a significant means of disseminating medical information (41).

### Strengths and Limitations

It should be noted, however, that this study has limitations. First, Twitter may not be entirely reflective of the general population. Second, researchers cannot measure behavior or clinical outcomes directly from tweets. Third, the dataset with 477 tweets is not large, and 101 tweets were eliminated because of limited information and unrelated content. Fourth, the utilization of codebook and text analysis implies a degree of subjectivity. However, this methodology is consistent with previous medical research studies on Twitter and could be applied to different topics and carried out by other research groups (42). Furthermore, to address this issue, the study comprised a series of steps of initial review, the design of a codebook, and the testing of a coder agreement. Although computerized machine-learning methods have been tested to automatically identify and classify topics in medical research using social media, we employed an analytical strategy based on the clinical expertise of the raters, which constituted a qualitative advantage in relation to automatized strategies (43). Finally, the content analysis of tweets included comparisons between different categories (e.g., non-surgical and surgical therapy, medical and non-medical contents, and pre- and post-menopause) that enabled us to make inferences from the text in order to summarize the content.

## Conclusions

To our knowledge, this project is the first to study the posts of major U.S. media outlets in regard to women's health in Twitter. Understanding the public view of these health issues is useful to better appraise the perceived demands for clinical care related to women's health. It could also help to better design health promotion initiatives and awareness strategies that include contents of interest to social media users and to stimulate communication between health care providers and patients. Although this study is focused on contraception, sexual dysfunction, infertility, and bone health, these results provide relevant information that probably can be applicable to other health issues. The involvement of health institutions in related conversations through social media appears to be desirable given the interest raised by medical contents posted in social media.

## Data Availability Statement

The raw data supporting the conclusions of this article will be made available by the authors, without undue reservation.

## Author Contributions

MA-M participated as principal contributor in the research design, manuscript writing, and submission. MA-M and ML-V participated in the content analysis and review. CD-V conducted and reported statistical analysis. CL and AG participated in the manuscript writing and review. MA-M and MM-G contributed as supervisors of all the stages. All authors contributed to the article and approved the submitted version.

## Conflict of Interest

The authors declare that the research was conducted in the absence of any commercial or financial relationships that could be construed as a potential conflict of interest.
